# Neonatal extracorporeal membrane oxygenation in Colombia: outcomes, complication burden, and circuit-level constraints in a 15-year single-center cohort

**DOI:** 10.3389/fped.2026.1884777

**Published:** 2026-09-04

**Authors:** Angelica Ortiz-Córdoba, Edgar Fabián Manrique-Hernández, Andrés Felipe Rubio-Duarte, Claudia C. Colmenares-Mejía, Juliana Ballesteros-Trillos, Jorge Alvarado-Socarras, Claudia Ximena Flórez-Rodríguez, Alvaro Durán-Hernández, Juan Pablo Otoya-Castrillón, Leonardo Salazar

**Affiliations:** 1 Congenital and Pediatric Heart Diseases Center, Cardiovascular Institute, Hospital Internacional de Colombia HIC - Fundación Cardiovascular de Colombia FCV - Fundación Universitaria FCV, Santander, Colombia; 2 ECMO and ventricular Assistance Program, Cardiovascular Institute, Hospital Internacional de Colombia HIC - Fundación Cardiovascular de Colombia FCV - Fundación Universitaria FCV, Santander, Colombia; 3 Pediatric Cardiac Intensive Care Unit, Cardiovascular Institute, Hospital Internacional de Colombia HIC - Fundación Cardiovascular de Colombia FCV - Fundación Universitaria FCV, Santander, Colombia; 4 Epidemiology and Data Analysis Unit, Hospital Internacional de Colombia HIC - Fundación Cardiovascular de Colombia FCV - Fundación Universitaria FCV, Santander, Colombia; 5 Research Institute, Hospital Internacional de Colombia HIC - Fundación Cardiovascular de Colombia FCV - Fundación Universitaria FCV, Santander, Colombia; 6 Neonatal Intensive Care Unit, Cardiovascular Institute, Hospital Internacional de Colombia HIC - Fundación Cardiovascular de Colombia FCV - Fundación Universitaria FCV, Santander, Colombia; 7 Cardio-Thoracic Surgery Department, Heart and Vascular Centre, Maastricht University Medical Centre (MUMC+), Maastricht, Netherlands

**Keywords:** critical care, extracorporeal membrane oxygenation, infant, Latin America, neonates & life cycle, respiratory insufficiency

## Abstract

**Background:**

Neonatal extracorporeal membrane oxygenation (ECMO) is a highly specialized therapy for severe respiratory and/or cardiovascular failure, but data from low- and middle-income countries remain limited. We aimed to describe outcomes, complications, and implementation-related challenges in a 15-year neonatal ECMO cohort from a tertiary referral center in Colombia.

**Methods:**

We conducted a retrospective single-center cohort study including neonates younger than 28 days who underwent a single ECMO run between 2010 and 2025. Data were obtained from an institutional dataset derived from the Extracorporeal Life Support Organization registry. ECMO configuration, indication, support duration, complications, and in-hospital mortality were analyzed descriptively. ECMO support was delivered using adapted centrifugal-pump circuits and polymethylpentene oxygenators within a multidisciplinary program in a middle-income setting.

**Results:**

Seventy neonates were included. ECMO support was predominantly venoarterial (68/70, 97.1%). Congenital heart disease was the most common diagnostic category (31/70, 44.3%). Overall in-hospital mortality was 47.1%. Overall ECMO median duration of 120 h (interquartile range, 76–217). At least one complication occurred in 40 patients (57.1%), the most frequent complications were renal replacement therapy (12.9%), cardiopulmonary resuscitation events (12.9%), circuit thrombosis (8.6%), and pulmonary hemorrhage (7.1%). Longer ECMO duration was associated with both complications and mortality.

**Conclusions:**

Neonatal ECMO can be delivered in a middle-income country within a structured multidisciplinary program. Outcomes appear influenced not only by clinical severity, but also by circuit-related constraints, hemocompatibility challenges, monitoring capacity, and resource-adapted care models.

## Introduction

Extracorporeal membrane oxygenation (ECMO) is an advanced form of temporary cardiopulmonary support used in neonates with severe respiratory and/or cardiovascular failure refractory to optimized conventional therapy. By providing extracorporeal gas exchange and circulatory support, ECMO may allow time for pulmonary, myocardial, or cardiorespiratory recovery. However, neonatal ECMO remains a high-risk intervention requiring specialized technology, systemic anticoagulation, and continuous multidisciplinary surveillance (1).

International registry data provide the main benchmark for neonatal ECMO outcomes, although survival varies substantially according to indication, physiology, mode of support, and associated organ dysfunction (2). Survival is generally highest among neonates supported for isolated respiratory failure and lower among those with cardiac disease or ECMO initiated during cardiopulmonary resuscitation, reflecting differences in baseline severity and disease reversibility. Contemporary ELSO data continue to demonstrate the global relevance of ECMO benchmarking, but registry-based estimates may not fully capture local differences in case mix, referral pathways, program structure, available technology, and care delivery models.

Neonatal ECMO management is particularly challenging because small circulating blood volume, developmental hemostasis, systemic anticoagulation (3), and exposure of blood to artificial circuit surfaces converge to create a narrow margin between bleeding, thrombosis, hemolysis, and circuit dysfunction. Bleeding and thrombotic events remain among the most frequent and clinically relevant complications of neonatal ECMO and are consistently associated with morbidity and mortality. Importantly, these events are influenced not only by patient severity and anticoagulation strategy, but also by circuit configuration, pump characteristics, tubing diameter, pressure-flow relationships, and the ability to monitor circuit health over time (4, 5).

These considerations are especially relevant in low- and middle-income countries, where ECMO programs often operate within structural constraints that differ from those of high-income, high-volume centers. In Latin America, neonatal and pediatric ECMO has expanded through selected referral centers and regional collaboration (6), but published data remain limited. Previous regional experience has shown that ECMO can be implemented successfully in South American programs despite financial, technical, and human-resource constraints (7). Nevertheless, there is still a need to understand how these constraints shape complications, outcomes, and opportunities for quality improvement.

Reports from individual centers in resource-constrained settings are therefore valuable not only for benchmarking survival, but also for identifying modifiable determinants of ECMO performance. We conducted a retrospective cohort study of neonates supported with ECMO at a tertiary referral center in Colombia to describe clinical characteristics, ECMO configuration, complication burden, and in-hospital outcomes. We also sought to interpret these findings within the context of program structure and circuit-level constraints encountered during neonatal ECMO delivery in a middle-income setting.

## Methods

### Study design and data source

We conducted a retrospective single-center cohort study including all neonates who underwent extracorporeal membrane oxygenation (ECMO) between 2010 and 2025 at a tertiary referral center in Colombia. Neonates were defined as patients younger than 28 days of age at the time of ECMO initiation. All consecutive neonates who underwent a single ECMO run during the study period were included. For patients with more than one ECMO run during the same hospitalization, only the first ECMO episode was considered. No additional exclusion criteria were applied.

Data were obtained from an institutional dataset derived from the Extracorporeal Life Support Organization (ELSO) registry, which prospectively captures standardized clinical, procedural, complication, and outcome variables for patients receiving ECMO support.

### Clinical variables and definitions

Baseline clinical characteristics included sex, gestational age, weight at ECMO cannulation, primary diagnosis, pre-ECMO ventilatory management (high-frequency oscillatory ventilation and inhaled nitric oxide), arterial blood gas measurements (pH, PaO₂, and PaCO₂), serum lactate, and pre-ECMO vasoactive support. Diagnoses were grouped into major clinical categories, including congenital diaphragmatic hernia, congenital heart disease, persistent pulmonary hypertension of the newborn, acute respiratory failure, and other diagnoses. ECMO indications were further classified as postcardiotomy cardiac failure, cardiogenic shock, refractory hypoxemia, pulmonary hypertension, and extracorporeal cardiopulmonary resuscitation (ECPR). Outcome variables included survival to decannulation, in-hospital mortality, death during ECMO support, death after decannulation, 28-day mortality, time from ECMO initiation to death, and cause of death.

ECMO configuration was classified as venoarterial (VA) or venovenous (VV). Extracorporeal cardiopulmonary resuscitation (ECPR) was categorized separately as the clinical context of ECMO initiation when support was established during or immediately after cardiopulmonary resuscitation. For descriptive analyses, ECMO indication was also categorized as primarily cardiac or primarily pulmonary according to the underlying disease process and the main physiological indication for support.

Complications were defined according to standardized ELSO registry criteria and categorized into major domains, including mechanical, hemorrhagic, neurologic, renal, cardiovascular, pulmonary, metabolic, and limb-related events. Mechanical complications included circuit thrombosis, oxygenator dysfunction, and circuit change when recorded. Complication burden was evaluated both as a binary variable, defined as at least one recorded complication versus none, and as the total number of recorded complications per patient.

The primary outcome was in-hospital mortality, defined as death from any cause before hospital discharge. Secondary outcomes included ECMO duration in hours and complication burden.

### ECMO management

ECMO support was delivered according to institutional protocols aligned with contemporary international recommendations and adapted to locally available resources. Indications for ECMO initiation included severe respiratory and/or cardiovascular failure refractory to optimized conventional management, including invasive mechanical ventilation, vasoactive support, and adjunctive therapies when indicated. Patient selection and timing of cannulation were determined by a multidisciplinary team, considering disease reversibility, clinical trajectory, expected benefit, and the absence of major contraindications. ECMO support was provided by a dedicated multidisciplinary team available 24 h a day, 7 days a week. Bedside care was maintained with a 1:2 ECMO specialist nurses-to-patient ratio, while continuous ECMO circuit coverage was ensured by ECMO specialists, including ECMO-trained physicians and ECMO specialist nurses. At our institution, ECMO specialist nurses are specifically trained in circuit priming, circuit preparation, continuous circuit monitoring, and first-line troubleshooting, working in close collaboration with perfusionists and the attending ECMO physician. Perfusionists were present during ECMO cannulation and remained available throughout the ECMO run for circuit-related support and troubleshooting.

Cannulation strategy and ECMO configuration were individualized according to patient physiology and anatomical considerations. VA-ECMO was the predominant modality, reflecting the high prevalence of combined cardiopulmonary failure, complex congenital heart disease, pulmonary hypertension, and hemodynamic instability in this cohort. Cannulation was typically performed via the right internal jugular vein and carotid artery. VV-ECMO was reserved for selected cases of isolated respiratory failure with preserved cardiac function. In addition to patient physiology, ECMO configuration was also influenced by local technical constraints. During the study period, the limited availability of neonatal VV-ECMO cannulas restricted the use of venovenous support in patients requiring isolated respiratory support. Therefore, ECMO configuration in this cohort should be interpreted in the context of both clinical characteristics and local resource availability. ECMO circuits were configured using Medtronic Affinity CP™ centrifugal pumps and LivaNova Lilliput™ ECMO polymethylpentene oxygenators. Because the available centrifugal pump platform has a 3/8-inch outlet and neonatal ECMO support requires smaller-diameter tubing and cannulation interfaces, circuit size transitions to 1/4-inch components were required. To mitigate low-flow or stagnant segments created by these size transitions, a 1/8-inch bridge between arterial and venous Luer access points was systematically incorporated into the circuit configuration. This adaptation was used in all neonatal ECMO cases during the study period.

Both circuit flow and patient-delivered flow were monitored. Circuit surveillance included visual inspection and intermittent assessment of circuit pressures. Continuous or systematic recording of pre- and post-oxygenator pressures was not performed as part of routine care throughout the study period. Instead, circuit pressures were assessed intermittently, particularly when changes in delivered flow occurred despite stable pump revolutions, in order to evaluate increased circuit resistance, oxygenator dysfunction, or other mechanical causes of impaired flow.

During ECMO support, blood flow and sweep gas were titrated to achieve adequate systemic perfusion and gas exchange, guided by clinical assessment, arterial blood gases, lactate levels, venous oxygen saturation when available, and markers of end-organ perfusion. A lung-protective ventilation strategy was used during ECMO, aiming to minimize ventilator-induced lung injury while allowing cardiopulmonary recovery. Pressure-controlled ventilation was the preferred mode whenever feasible. Peak inspiratory pressure was generally maintained below 25 cmH₂O, with plateau pressure limited to 25–28 cmH₂O, PEEP between 8 and 12 cmH₂O, respiratory rates of 10–17 breaths/min, and ventilator FiO₂ between 30% and 60%. When reliably measurable, tidal volumes were maintained below 4 mL/kg. Ventilator settings were not standardized to fixed targets but were individualized according to the ECMO configuration, patient age, the severity and etiology of lung injury, respiratory-system compliance, gas exchange requirements, and hemodynamic status. High-frequency oscillatory ventilation was used selectively, at the discretion of the treating team, in patients with severe respiratory failure when clinically indicated.

Systemic anticoagulation was maintained with unfractionated heparin. Heparin dosing was adjusted primarily according to activated partial thromboplastin time, measured every 6 h and repeated as clinically indicated in the presence of bleeding or suspected circuit thrombosis. Anticoagulation monitoring also incorporated activated clotting time and anti-factor Xa activity when available, together with platelet count, fibrinogen concentration, routine coagulation parameters, and continuous clinical and circuit assessment. During the study period, point-of-care coagulation monitoring, including activated clotting time and thromboelastography, was progressively incorporated to optimize anticoagulation and guide transfusion therapy. Anticoagulation and hemostatic management were individualized to balance thrombotic and hemorrhagic complications. Additional organ support, including continuous kidney replacement therapy, was provided as clinically indicated. The bedside care model consisted of a trained nurse responsible for simultaneous patient care and circuit surveillance, with support from the multidisciplinary ECMO team according to institutional protocols. This model informed a pragmatic monitoring strategy focused on continuous bedside clinical assessment, visual circuit surveillance, flow monitoring, laboratory monitoring, and targeted pressure assessment when clinically indicated.

Weaning from ECMO was considered upon evidence of cardiopulmonary recovery, using stepwise flow reduction and repeated clinical reassessment. Decannulation was performed after multidisciplinary consensus. All cases were recorded in a structured database using standardized definitions to support quality monitoring and benchmarking.

### Statistical analysis

Continuous variables were summarized as medians with interquartile ranges (IQR), given non-normal distributions. Categorical variables were reported as counts and percentages. Analyses of ECMO duration were restricted to patients with available data, and missing data were handled using a complete-case approach without imputation. Given the descriptive nature of the study and the limited sample size, no multivariable modeling was performed. Analyses were conducted overall and descriptively according to ECMO configuration, clinical indication, complication burden, and survival status. All analyses were performed using Stata software.

### Ethical considerations

The study was approved by the Institutional Ethics Committee of the Fundación Cardiovascular de Colombia (approval number CEI-2024-07461-41). The requirement for informed consent was waived owing to the retrospective nature of the study.

## Results

A total of 70 neonates were included in the analysis. Most patients were male (74.3%). ECMO support was predominantly venoarterial (VA) (97.1%), whereas venovenous (VV) ECMO was used in 2 patients (2.9%). The most frequent primary diagnoses were congenital heart diseases (31 [44.29%]) and acute respiratory failure (8 [11.43%]). Overall in-hospital mortality was 47.14% (Table 1).

**Table 1:** Baseline characteristics of the study population.

Characteristics	*n* = 70	
	*n*	%
Category		
Male	52	74.29
Female	18	25.71
ECMO mode		
VA	68	97.14
VV	2	2.86
Diagnosis		
Congenital diaphragmatic hernia	7	10.00
Congenital heart diseases	31	44.29
Acute respiratory failure	8	11.43
Pulmonary hypertension of newborn	6	8.57
Other	18	25.71
In-hospital mortality	33	47.14

VA, venoarterial; VV, venovenous.

ECMO duration was available for 67 patients. The median duration of support was 120 h (interquartile range [IQR], 76–217). Duration differed according to ECMO modality, with longer runs observed in VV ECMO (median, 217 h [IQR, 120–384]) than in VA ECMO (median, 106 h [IQR, 74–168]). The single patient who underwent extracorporeal cardiopulmonary resuscitation (ECPR) had a duration of 89 h (Table 2). Additional baseline clinical characteristics are summarized in Table 2. The cohort consisted predominantly of term neonates, with a median gestational age ranging from 38.0 to 40.0 weeks and a median birth weight of approximately 3.1–3.3 kg across ECMO indications. High-frequency oscillatory ventilation and inhaled nitric oxide were used primarily in the pulmonary ECMO group, whereas epinephrine was the most frequently used pre-ECMO vasoactive agent. Twenty-eight-day mortality and the timing and causes of death are also summarized in Table 3.

**Table 2:** ECMO duration according to clinical characteristics and outcomes.

Characteristics	*n*	Median (IQR), hours
Support type		
Cardiac	53	106.17 (74–168)
ECPR	1	89 (89–89)
Pulmonary	13	217 (120–384.43)
Complications		
No complications	30	88 (48–168)
≥1 complication	40	124.5 (89–255)
Live status at discharge		
Alive	36	96.18 (69.91–144.55)
Dead	31	168 (86–301)

ECPR, extracorporeal cardiopulmonary resuscitation.

**Table 3:** Baseline clinical characteristics, ECMO indications, and outcomes according to ECMO support type.

Characteristics	Cardiac (*n* = 54)	Pulmonary (*n* = 15)	ECPR (*n* = 1)
Type ECMO			
Gestational age, weeks	40.00 (38.52–40.83)	38.00 (37.15–38.45)	39.2
Weight at ECMO cannulation, kg	3.30 (3.10–3.70)	3.10 (2.95–3.20)	3.2
Pre-ECMO ventilatory management			
HFOV	12 (28.6%)	8 (72.7%)	0 (0.0%)
Inhaled nitric oxide	5 (11.9%)	8 (72.7%)	0 (0.0%)
Pre-ECMO arterial blood gas			
pH	7.32 (7.20–7.37)	7.11 (7.04–7.20)	7.12
PaO₂ (mmHg)	45.00 (32.75–52.45)	29.20 (21.75–31.65)	145
PaCO₂ (mmHg)	51.75 (40.25–61.88)	61.90 (60.00–80.75)	31.5
Serum lactate, mmol/L	2.30 (1.66–6.30)	2.30 (1.48–2.92)	6
Pre-ECMO vasoactive support			
Epinephrine	20 (100.0%)	5 (100.0%)	1 (100.0%)
Milrinone	19 (45.2%)	1 (9.1%)	0 (0.0%)
Vasopressin	3 (7.1%)	3 (27.3%)	0 (0.0%)
Dopamine	3 (7.1%)	5 (45.5%)	1 (100.0%)
Dobutamine	3 (7.1%)	1 (9.1%)	0 (0.0%)
Prostaglandin E1	14 (33.3%)	0 (0.0%)	0 (0.0%)
Norepinephrine	6 (14.3%)	4 (36.4%)	0 (0.0%)
ECMO indication			
ECPR	0 (0.0%)	0 (0.0%)	1 (100.0%)
Severe ventricular dysfunction following cardiac arrest	1 (2.4%)	0 (0.0%)	0 (0.0%)
Cardiogenic shock	8 (19.0%)	3 (27.3%)	0 (0.0%)
Pulmonary hypertension	2 (4.8%)	1 (9.1%)	0 (0.0%)
Refractory hypoxemia	6 (14.3%)	6 (54.5%)	0 (0.0%)
Failure to wean from cardiopulmonary bypass (postcardiotomy)	25 (59.5%)	1 (9.1%)	0 (0.0%)
Outcomes			
In-hospital death	23 (42.6%)	9 (60.0%)	1 (100.0%)
Death during ECMO support	3 (42.9%)	4 (80.0%)	1 (100.0%)
Death after decannulation	4 (57.1%)	1 (20.0%)	0 (0.0%)
Time from ECMO initiation to death, days	24.50 (13.75–38.50)	10.50 (6.25–15.50)	6
28-day mortality post decannulation	7 (18.4%)	3 (33.3%)	0 (0.0%)
Cause of death			
Multiple organ dysfunction syndrome	2 (11.1%)	3 (50.0%)	0 (0.0%)
Neurological injury	6 (33.3%)	2 (33.3%)	1 (100.0%)
Persistent cardiovascular failure	5 (27.8%)	0 (0.0%)	0 (0.0%)
Pulmonary embolism	1 (5.6%)	0 (0.0%)	0 (0.0%)
Sepsis	4 (22.2%)	0 (0.0%)	0 (0.0%)
ECMO-related complications	0 (0.0%)	1 (16.7%)	0 (0.0%)

Data are presented as median (interquartile range [IQR]) or *n* (%), as appropriate.

ECPR, extracorporeal cardiopulmonary resuscitation; ECMO, extracorporeal membrane oxygenation; HFOV, high-frequency oscillatory ventilation; PaO₂, arterial partial pressure of oxygen; PaCO₂, arterial partial pressure of carbon dioxide. Baseline clinical variables were unavailable for 16 patients because of incomplete retrospective documentation and are reported as missing in the corresponding table entries.

At least one complication occurred in 40 patients (57.1%), whereas 30 (42.9%) had no recorded complications. Patients with complications had longer ECMO runs than those without complications (median, 124.5 h [IQR, 89–255] vs. 88 h [IQR, 48–168]). Similarly, non-survivors had longer ECMO duration than survivors (median, 168 h [IQR, 86–301] vs. 96.2 h [IQR, 69.9–144.6]). The most frequent complications were renal replacement therapy (9 [12.9%]), cardiopulmonary resuscitation events (9 [12.9%]), circuit thrombosis (6 [8.6%]), and pulmonary hemorrhage (5 [7.1%]). The incidence of all ECMO-related complications, classified according to the standardized ELSO definitions, is presented in Table 4.

**Table 4:** Frequency of ECMO-related complications according to standardized ELSO classification.

ECMO-related complications	*n*	%
Complication		
Renal: Renal Replacement Therapy Required	9	12.86
Cardiovascular: CPR	9	12.86
Mechanical: Thrombosis/Clots: circuit component	6	8.57
Pulmonary: Pulmonary hemorrhage	5	7.14
Mechanical: Circuit change	4	5.71
Mechanical: Cannula problems	3	4.29
Renal: Creatinine 1.5–3.0	3	4.29
Cardiovascular: Other event	3	4.29
Pulmonary: Pneumothorax requiring treatment	3	4.29
Other complications	3	4.29
Hemorrhagic: GI hemorrhage	2	2.86
Neurologic: CNS diffuse ischemia	2	2.86
Cardiovascular: Cardiac arrhythmia (other)	2	2.86
Mechanical: Oxygenator failure	1	1.43
Mechanical: Clots: hemofilter	1	1.43
Mechanical: Air in circuit	1	1.43
Neurologic: CNS Infarction (US/CT/MRI)	1	1.43
Neurologic: Intraventricular CNS hemorrhage	1	1.43
Cardiovascular: Cardiac arrhythmia	1	1.43
Cardiovascular: Tamponade (blood)	1	1.43
Metabolic: Hyperbilirubinemia	1	1.43
Metabolic: Moderate hemolysis	1	1.43
Metabolic: Severe hemolysis	1	1.43

CPR, cardiopulmonary resuscitation; GI, gastrointestinal; CNS, central nervous system; US, ultrasound; CT, computed tomography; MRI, magnetic resonance imaging.

**Figure 1:**
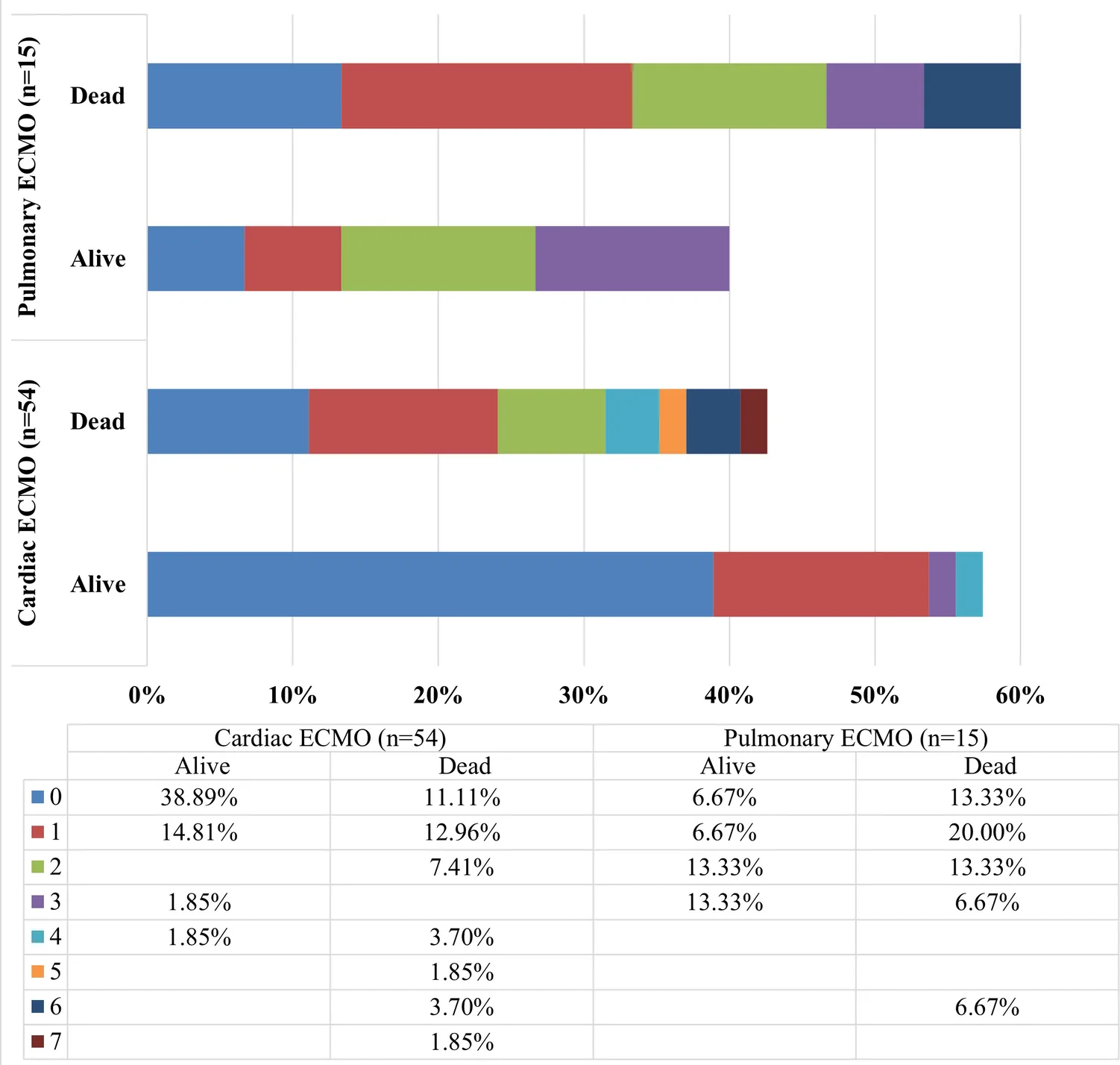
In-hospital mortality according to number of complications and ECMO modality. ECPR, extracorporeal cardiopulmonary resuscitation.

When stratified according to ECMO modality and complication burden, a graded increase in mortality was observed with a higher number of complications, particularly among patients supported with VA ECMO (Figure 1). Among patients receiving VA ECMO with no recorded complications, survival was 77.8%, whereas mortality increased progressively with increasing complication burden.

## Discussion

In this 15-year single-center cohort of neonates supported with extracorporeal membrane oxygenation in a middle-income country, overall survival to hospital discharge was approximately 53%. This outcome is clinically meaningful given the complexity of the cohort, which was characterized by a high proportion of neonates with congenital heart disease, predominant use of VA-ECMO, and frequent organ-support needs and complications. These findings support the feasibility of neonatal ECMO delivery in a resource-constrained setting when care is provided within a structured, experienced, multidisciplinary program. At the same time, they highlight that survival alone provides an incomplete picture of program performance, particularly in settings where patient severity, device availability, circuit configuration, monitoring capacity, and staffing models differ from those of high-income ECMO centers.

International registry data remain the main benchmark for neonatal ECMO outcomes (2), but comparisons across programs require careful interpretation. Neonatal ECMO survival varies substantially by indication, with generally better outcomes in isolated respiratory failure and lower survival among neonates with cardiac disease or ECMO initiated in the setting of cardiopulmonary resuscitation. In our cohort, the predominance of VA-ECMO and the high burden of complex cardiovascular disease likely influenced both the observed mortality and the complication profile. Thus, the overall survival observed in this study should be interpreted less as a direct comparison with neonatal respiratory ECMO benchmarks and more as a real-world outcome from a high-complexity referral population in Latin America.

The predominance of VA-ECMO in this series reflects neonatal physiology, referral patterns, and local resource availability. Critically ill neonates with respiratory failure frequently present with pulmonary hypertension, myocardial dysfunction, or hemodynamic instability, making VA-ECMO an attractive option because it provides both gas exchange and circulatory support. However, ECMO configuration was also influenced by local technical constraints. During the study period, the limited availability of neonatal VV-ECMO cannulas restricted the use of venovenous support. Consequently, VA-ECMO was used in selected patients with predominantly respiratory failure for whom VV-ECMO was not technically feasible. Therefore, ECMO configuration in this cohort should be interpreted in the context of both patient physiology and local resource availability.

A central finding of this study is the association of ECMO-related complications with longer ECMO duration and mortality. More than half of the patients experienced at least one recorded complication, and those who developed complications required longer ECMO support. This observation is consistent with previous neonatal and pediatric ECMO studies, in which bleeding, thrombosis, renal dysfunction, neurologic injury, and mechanical complications remain major contributors to ECMO-related morbidity (8, 9) Compared with the contemporary ELSO cohort reported by Khan et al. (10), gastrointestinal hemorrhage, severe hemolysis, diffuse CNS ischemia, and intraventricular hemorrhage were numerically more frequent in our cohort, whereas CNS infarction was less frequent (Table 3). These comparisons should be interpreted descriptively given the differences in patient populations and study design. Importantly, ECMO-related complications should not be viewed solely as consequences of baseline disease severity, but rather as the result of the complex interaction between patient physiology, anticoagulation, circuit hemocompatibility, surveillance practices, and program-level processes of care (3). This interaction is particularly relevant for interpreting circuit-related complications in our cohort. Our neonatal ECMO circuits were configured using Medtronic Affinity CP centrifugal pumps and LivaNova Lilliput ECMO polymethylpentene oxygenators. Because the available centrifugal pump platform has a 3/8-inch outlet, neonatal support required transitions to 1/4-inch components and cannulation interfaces. These transitions may create segments of relatively low flow velocity, increasing the potential for blood stasis, thrombus formation, and impaired hemocompatibility. To mitigate this risk, our program systematically incorporated a 1/8-inch bridge between arterial and venous Luer access points, aiming to increase local flow velocity in low-flow portions of the circuit. Although this adaptation is physiologically rational and has been incorporated as routine practice in our program, it also increases circuit complexity and does not fully reproduce the flow dynamics of a dedicated neonatal ECMO platform.

The relevance of this device-level constraint is supported by emerging neonatal ECMO literature. Vermeer et al. described hemostatic complications after transition from a roller-pump neonatal ECMO circuit to a centrifugal pump circuit with 3/8-inch inlet and outlet, including recurrent acute increases in oxygenator resistance requiring emergency changeout; after transition to a levitating centrifugal pump with 1/4-inch inlet and outlet, oxygenator malfunction was no longer observed in that report (11). The authors suggested that low blood velocities, recirculation near the centrifugal pump, and heat generation could contribute to clot formation. This experience is highly relevant to our setting because it illustrates that circuit geometry and pump-tubing compatibility may affect neonatal ECMO hemocompatibility independently of bedside expertise.

The broader literature comparing roller and centrifugal pumps in neonatal and pediatric ECMO remains heterogeneous. Some studies and meta-analyses have suggested lower mortality or fewer complications with roller pumps in selected neonatal and pediatric populations (12), whereas other registry-based analyses have produced more nuanced findings depending on ECMO mode, patient selection, and era of care (13). Therefore, our findings should not be interpreted as evidence that one pump type is universally superior. Rather, they emphasize that the clinical effect of pump technology depends on the entire circuit configuration, including tubing diameter, connectors, cannula size, pump design, flow requirements, and monitoring practices (14). For neonatal ECMO, where flows are low and circulating blood volume is small, even subtle areas of stasis or recirculation may have disproportionate clinical consequences (15).

Circuit surveillance is another key issue (5); in our program, both circuit flow and patient-delivered flow are monitored, and circuit pressures are assessed intermittently, particularly when delivered flow changes despite stable pump revolutions. Continuous systematic recording of pre- and post-oxygenator pressures was not part of routine care throughout the study period. This practice reflects a pragmatic balance between ideal circuit monitoring and the realities of bedside workload in a resource-constrained environment. Recent neonatal ECMO studies have shown that circuit health may be predicted by changes in delta pressure, pump revolutions, plasma-free hemoglobin, and other functional parameters before circuit impairment or circuit change (16). These findings suggest that future quality-improvement efforts in our program should prioritize more systematic circuit-health monitoring, while ensuring that such monitoring is feasible within the local staffing model.

The bedside care model also deserves specific consideration. Our program uses trained nurses as frontline ECMO caregivers, with support from intensivists, surgeons, perfusionists, respiratory therapists, and the broader multidisciplinary ECMO team. This model has been central to the feasibility and sustainability of ECMO in our setting and is aligned with previous South American experience showing that nurse-based ECMO specialist models can be implemented successfully in developing countries (7). However, when the same bedside nurse is responsible for both patient care and circuit surveillance, monitoring strategies must be designed to reduce workload, avoid alarm fatigue, and preserve safety. This is not simply a staffing detail; it is a systems-level determinant of how ECMO is delivered, monitored, and improved in middle-income settings.

From a health-system perspective, these findings reinforce the need to interpret ECMO outcomes in Latin America through a broader lens than survival alone (17). Programs in resource-constrained settings often operate with limited access to neonatal-specific ECMO technology, variable referral timing, constrained staffing, and financial limitations (17, 18). Despite these barriers, neonatal and pediatric ECMO has expanded in selected Latin American referral centers, and prior regional experience has shown that advanced extracorporeal support can be implemented successfully when programs are centralized, protocolized, and multidisciplinary. Our findings add to this literature by showing not only that neonatal ECMO is feasible in Colombia, but also that outcomes are shaped by the interface between technology, staffing, monitoring capacity, and patient complexity.

This study has several limitations. First, its retrospective, single-center design may limit the generalizability of the findings. Second, despite the inclusion of additional baseline clinical variables, including gestational age, birth weight, pre-ECMO lactate, arterial blood gas measurements, vasoactive support, decannulation survival, and 28-day mortality, the retrospective nature of the study precluded the systematic collection of validated illness severity scores, vasoactive burden indices, detailed anticoagulation parameters, and longitudinal lactate trajectories across the entire study period. Third, although circuit configuration and monitoring practices are described, circuit pressures, delta-pressure trends, plasma-free hemoglobin, and standardized reasons for circuit change were not consistently captured throughout the study period. Finally, long-term neurodevelopmental and functional outcomes were not available, precluding assessment of the impact of ECMO beyond hospital discharge. Despite these limitations, this study contributes important data from a region that remains underrepresented in the neonatal ECMO literature. It also identifies specific opportunities for quality improvement: systematic collection of circuit-health metrics, standardized classification of adverse events, prospective tracking of circuit changes and oxygenator performance, refinement of anticoagulation monitoring, and evaluation of staffing models that balance safety with feasibility. Future multicenter collaboration across Ibero-America would allow risk-adjusted benchmarking, shared learning, and evaluation of whether access to neonatal-optimized circuits improves hemocompatibility, complication burden, and survival.

In conclusion, neonatal ECMO can be delivered successfully in a middle-income country within a structured, high-expertise program. However, our experience suggests that outcomes in such settings are shaped not only by clinical severity and program organization, but also by device-level constraints and pragmatic models of care. Recognizing this technology–implementation gap is essential for fair benchmarking, sustainable program development, and quality improvement in Latin America and other resource-constrained regions.

## Conclusion

Neonatal ECMO can be delivered successfully in a middle-income country when care is centralized within a structured, experienced, multidisciplinary program. In this 15-year single-center cohort, survival was clinically meaningful despite a high-complexity case mix characterized by predominant VA-ECMO use and a substantial proportion of neonates with congenital heart disease and cardiovascular compromise.

Complication burden was closely associated with longer ECMO duration and mortality, underscoring the importance of adverse-event prevention, early recognition, and systematic quality improvement during extracorporeal support. In our setting, complications should be interpreted within the interaction between patient severity, anticoagulation management, circuit hemocompatibility, device-level constraints, and bedside monitoring capacity.

Beyond demonstrating feasibility, our experience highlights a technology–implementation gap that is particularly relevant for neonatal ECMO in resource-constrained settings. The use of adapted circuits rather than fully neonatal-optimized ECMO platforms may affect flow dynamics, circuit complexity, and hemocompatibility, while pragmatic staffing and monitoring models shape how circuit surveillance is performed at the bedside.

Future efforts should focus on prospective collection of circuit-health metrics, standardized reporting of complications, refinement of anticoagulation and monitoring protocols, and multicenter collaboration across Ibero-America. These strategies are essential to improve risk-adjusted benchmarking, guide quality improvement, and support the safe, equitable, and sustainable expansion of neonatal ECMO in Latin America and similar health-system contexts.

## Data Availability

The raw data supporting the conclusions of this article will be made available by the authors, without undue reservation.
